# Supplementary material for: Untargeted lipidomics analysis in women with morbid obesity and type 2 diabetes mellitus: A comprehensive study
Source: PLoS One. 2024 May 14;19(5):e0303569. doi: 10.1371/journal.pone.0303569 (PMC11093320; doi:10.1371/journal.pone.0303569)
Supplement: Attachment — Submitted filename: Response to Reviewers ok.pdf [file pone.0303569.s004.pdf]

RESPONSE TO EDITOR AND REVIEWERS

Firstly, esteemed editorial board of the journal and reviewers, we greatly appreciate the opportunity to correct and improve our manuscript with your review and your comments and suggestions. We will address all your comments one by one, replying on this same response letter, marking our response in blue.

Journal Requirements:

1. Please ensure that your manuscript meets PLOS ONE's style requirements, including those for file naming. The PLOS ONE style templates can be found at "https://journals.plos.org/plosone/s/file?id=wjVg/PLOSOne_formatting_sample_main_body.pdf" and "https://journals.plos.org/plosone/s/file?id=ba62/PLOSOne_formatting_sample_title_authors_affiliations.pdf"

Answer 1: Following the guidelines you specified, in the revised version of our manuscript, we have adapted the affiliations and the entire text to the recommended style. The changes have been marked with track changes in the document.

2. We note that the grant information you provided in the 'Funding Information' and 'Financial Disclosure' sections do not match. When you resubmit, please ensure that you provide the correct grant numbers for the awards you received for your study in the 'Funding Information' section.

Answer 2: Alright, we will review this information when uploading the article. The project code specified in the manuscript belongs to an internally funded project of the group (Fundació URV Project number IT20041-S to CR), as no funds have been obtained from other institutions or competitive grants for the completion of this study.

3. Thank you for stating the following in the Acknowledgments Section of your manuscript:

"Thanks to the Universitat Rovira i Virgili for its administrative collaboration and Fundació URV for its collaboration in the funding of this study (Project IT20041-S to C.R)."

We note that you have provided funding information that is not currently declared in your Funding Statement. However, funding information should not appear in the Acknowledgments section or other areas of your manuscript. We will only publish funding information present in the Funding Statement section of the online submission form.

Please remove any funding-related text from the manuscript and let us know how you would like to update your Funding Statement. Currently, your Funding Statement reads as follows:

"The authors received no specific funding for this work."

Please include your amended statements within your cover letter; we will change the online submission form on your behalf.

Answer 3: Again, it should be noted that the project from the Fundació URV mentioned in the acknowledgments does not come from external institutions, and therefore, we have not received funding for the realization of this project. Instead, it has been financed with internal funds. In this regard, the phrase "The authors received no specific funding for this work." would be correct in the submission platform. On the other hand, we have removed all funding information from the Acknowledgments section in the revised version of our manuscript.

4. We note that your Data Availability Statement is currently as follows: [All relevant data are within the manuscript and its Supporting Information files.]

Please confirm at this time whether or not your submission contains all raw data required to replicate the results of your study. Authors must share the “minimal data set” for their submission. PLOS defines the minimal data set to consist of the data required to replicate all study findings reported in the article, as well as related metadata and methods (https://journals.plos.org/plosone/s/data-availability#loc-minimal-data-set-definition).

For example, authors should submit the following data:

- The values behind the means, standard deviations and other measures reported;

- The values used to build graphs;

- The points extracted from images for analysis.

Authors do not need to submit their entire data set if only a portion of the data was used in the reported study.

If your submission does not contain these data, please either upload them as Supporting Information files or deposit them to a stable, public repository and provide us with the relevant URLs, DOIs, or accession numbers. For a list of recommended repositories, please see https://journals.plos.org/plosone/s/recommended-repositories.

If there are ethical or legal restrictions on sharing a de-identified data set, please explain them in detail (e.g., data contain potentially sensitive information, data are owned by a third-party organization, etc.) and who has imposed them (e.g., an ethics committee). Please also provide contact information for a data access committee, ethics committee, or other institutional body to which data requests may be sent. If data are owned by a third party, please indicate how others may request data access.

Answer 4: We confirm that all the raw data necessary to replicate our results was already included in the Supporting Information files (Tables S1 and S2).

5. Please include your full ethics statement in the ‘Methods’ section of your manuscript file. In your statement, please include the full name of the IRB or ethics committee who approved or waived your study, as well as whether or not you obtained informed written or verbal consent. If consent was waived for your study, please include this information in your statement as well.

Answer 5: In the manuscript, in the Materials and Methods section (lines 104-105, page 5), the requested information is already provided. It includes the full name of the ethics committee, the study code approved by the ethics committee, and it specifies that all patients provided written informed consent to participate in this study.

6. Please review your reference list to ensure that it is complete and correct. If you have cited papers that have been retracted, please include the rationale for doing so in the manuscript text, or remove these references and replace them with relevant current references. Any changes to the reference list should be mentioned in the rebuttal letter that accompanies your revised manuscript. If you need to cite a retracted article, indicate the article’s retracted status in the References list and also include a citation and full reference for the retraction notice.

Answer 6: We have updated the reference list and can confirm that it adheres to the format requested by the journal.

Reviewers' comments:

Reviewer #1:

• In the methodology section authors say that "The study cohort of this study was composed by only women to evaluate a homogenous cohort of subjects to avoid the interference of confounding factors such as sex". This is a valid point. However, authors need to consider several other confounding factors, mainly statin users between the groups. Other factors that could affect serum lipid levels include alcohol intake and smoking. Also, there is no information on whether these patients are hypertensive.

Answer: First of all, thank you for your valuable feedback, which has greatly contributed to the refinement of our manuscript.

We thoroughly understand your comment and wholeheartedly agree with your observation. Our cohort of women in this study is derived from a collection of patients/samples, predominantly comprising women, with fairly specific inclusion and exclusion criteria. It is indeed true that we avoided including patients with an alcohol consumption exceeding 10g per day, as well as those with a history of recurrent tobacco use, as these factors can evidently impact metabolic parameters. Upon verifying these data, we confirm that all patients meet these criteria. Therefore, in the Materials and Methods section, under exclusion criteria, we have added the following phrase (lines 112-114, pages 5-6): "In addition, women with an alcohol intake exceeding 10g per day and recurrent smokers have been excluded from this study."

Furthermore, regarding the use of statins, we were unable to verify this information as we only have access to whether patients are taking lipid-lowering agents (not statins or fibrates). As discussed in the Results section (line 241, page 11), 28% of our cohort (comprising morbidly obese individuals) are taking this type of medication. Consequently, we have included this information again in the Limitations section (line 505-507, page 24): "Moreover, the 28% of subjects with morbid obese were treated with lipid-lowering agents, which could affect the obtained lipidome."

Lastly, we would like to note that we have not included blood pressure data in Tables 1 and 2. Although 39.8% of the patients suffer from hypertension, 98% of them are taking medication for high blood pressure. Therefore, no differences in blood pressure were observed among the different study groups.

• Correlation analysis between key parameters could better help to identify the linking of specific lipids with other parameters.

Answer: We agree with your suggestion and have added a section at the end of the results section with the correlation analysis of all lipid metabolites with various biochemical and anthropometric parameters of our study cohort (lines 327-341, page 17). Although these correlations have been discussed in the text, we have added a supplementary **table (S3 Table)** with all the correlations.

• Also, an additional table providing Anthropometric and biochemical data of the study subgroup one with T2DM (71) and the (n= 65) with metabolically healthy morbid obese cases. This table could essentially provide more insights.

Answer: In this regard, we already have included this table (Table 2). However, it is true that this table did not include anthropometric data such as age, BMI and waist-hip ratio. In this sense, we have included this information in the **new Table 2** (page 12).

• Article misses discussion from the important information on untargeted lipidomics data from prospective studies (For example: PMID: 35035379)

Answer: Dear reviewer, we agree with your suggestion and have added a paragraph in the discussion section (lines 347-353, page 18) where this article you suggested, along with two other related ones, is cited, defining the importance and applicability of these untargeted lipidomics studies in prospective cohorts: "In the context of obesity and T2DM, previous studies employing untargeted lipidomics in prospective cohorts have underscored the pivotal role of such investigations in biomarker development, understanding pathophysiology, personalized medicine tool development, therapeutic target identification, early disease detection, and population risk stratification [30–32]. These studies have illuminated the intricate lipidomic alterations associated with these metabolic conditions, offering invaluable insights into their etiology and progression."

Reviewer #2:

The article sent for evaluation is very interesting and fits into current scientific trends. The introduction is a very good introduction to the topic. Methodology - patients were correctly classified following the adopted assumptions. I was only wondering about the issue of menopause, which significantly affects metabolism. On the other hand, the average age in both groups is similar; hence, the share of women at menopausal age should be comparable. However, this does not exclude early menopause at the age of, for example, 40. If researchers do not have such information, it should be included in the limitation of the study section. The laboratory data lacked information on kidney function assessment, which significantly affects the body's lipid metabolism. I also found no information based on recruitment for the study, especially for women with normal body weight. What about comorbid conditions apart from diabetes mellitus?

Answer: First, thank you for your experienced feedback, which has greatly contributed to the improvement of our manuscript. Regarding your concern about menopause, I would like to point out that we did not recruit menopausal women or those with severe premenopausal symptoms, nor did we include patients using hormonal contraceptives or similar treatments. This was done to avoid hormonal and metabolic biases in our results, and this information was already detailed in the exclusion criteria of our study (lines 111-112, page 5).

As for the lack of information on the renal function of the patients, we acknowledge that this is an important limitation that should be mentioned in its corresponding section (lines 504-505, pages 24).

Lastly, I would like to highlight that although we have focused on diabetes mellitus due to its impact on the lipid profile of our patients, our cohort mainly consists of women with morbid obesity, many of whom suffer from other comorbidities such as dyslipidemia and hypertension. Regarding dyslipidemia, as detailed in limitations (lines 505-507, page 24), approximately 28% of the patients are under treatment for this condition, which practically represents all those with dyslipidemia in our obesity cohort (which constitutes 30% of the obese cohort).

Additionally, as mentioned earlier, approximately 39.8% of obese women suffer from hypertension, but the vast majority of them are under treatment. Therefore, it is possible that these diseases are "nullified" in terms of biochemical differences between the groups, as they remain within normal ranges, as described in the results (line 242-244, page 11).

Moreover, it's important to mention that both normal-weight patients and those with metabolically healthy morbid obesity do not suffer from any metabolic alterations.

Regarding the recruitment of patients, although we have defined that women with morbid obesity are part of patients undergoing bariatric surgery and have established the sample collection period and access to clinical data for the entire cohort, we have now added that women with normal weight are volunteers who meet the criteria for metabolic health (normal BMI and no defined metabolic alterations) (lines 97-98, page 5).

The discussion is interesting, but you can get lost, so I suggest the authors divide it into small sections.

Answer: I appreciate your comment and we have subdivided the Discussion section to make it more readable into:

- Lipidome comparative between morbidly obese and normal-weight women
- Lipidome comparative between morbidly obese with type 2 diabetes mellitus and metabolically healthy morbid obese women
- Lipidome of both comparatives
- Limitations

There are no applications; they need to be supplemented. I recommend adding a limitation to the study section. It would be helpful to have a diagram of the research, which was done one by one, so that the reader could sort it all out. A graphical abstract would also be helpful - where the authors would present their most important discovery. The two schemes I mentioned could be combined into one - it's up to the researchers to decide.

Answer: According to your suggestion, we have uploaded a graphical abstract/diagram of our study to make them easier to understand (file name: Graphical_abstract.tif).
